# A Conceptual Exploration of Hamstring Muscle–Tendon Functioning during the Late-Swing Phase of Sprinting: The Importance of Evidence-Based Hamstring Training Frameworks

**DOI:** 10.1007/s40279-023-01904-2

**Published:** 2023-09-05

**Authors:** Judd T. Kalkhoven, Mathias Lukauskis-Carvajal, Deborah L. Sides, Blake D. McLean, Mark L. Watsford

**Affiliations:** 1 Sport & Exercise Science Discipline Group, Faculty of Health, Human Performance Research Centre, University of Technology Sydney, Moore Park Precinct, PO Box 123, Broadway, NSW 2007 Australia; 2 SpeedLab, Cali, Colombia; 3 UK Sports Institute, Manchester Institute of Health and Performance, Manchester, UK

## Abstract

An eccentrically lengthening, energy-absorbing, brake-driven model of hamstring function during the late-swing phase of sprinting has been widely touted within the existing literature. In contrast, an isometrically contracting, spring-driven model of hamstring function has recently been proposed. This theory has gained substantial traction within the applied sporting world, influencing understandings of hamstring function while sprinting, as well as the development and adoption of certain types of hamstring-specific exercises. Across the animal kingdom, both spring- and motor-driven muscle–tendon unit (MTU) functioning are frequently observed, with both models of locomotive functioning commonly utilising some degree of active muscle lengthening to draw upon force enhancement mechanisms. However, a method to accurately assess hamstring muscle–tendon functioning when sprinting does not exist. Accordingly, the aims of this review article are three-fold: (1) to comprehensively explore current terminology, theories and models surrounding muscle–tendon functioning during locomotion, (2) to relate these models to potential hamstring function when sprinting by examining a variety of hamstring-specific research and (3) to highlight the importance of developing and utilising evidence-based frameworks to guide hamstring training in athletes required to sprint. Due to the intensity of movement, large musculotendinous stretches and high mechanical loads experienced in the hamstrings when sprinting, it is anticipated that the hamstring MTUs adopt a model of functioning that has some reliance upon active muscle lengthening and muscle actuators during this particular task. However, each individual hamstring MTU is expected to adopt various combinations of spring-, brake- and motor-driven functioning when sprinting, in accordance with their architectural arrangement and activation patterns. Muscle function is intricate and dependent upon complex interactions between musculoskeletal kinematics and kinetics, muscle activation patterns and the neuromechanical regulation of tensions and stiffness, and loads applied by the environment, among other important variables. Accordingly, hamstring function when sprinting is anticipated to be unique to this particular activity. It is therefore proposed that the adoption of hamstring-specific exercises should not be founded on unvalidated claims of replicating hamstring function when sprinting, as has been suggested in the literature. Adaptive benefits may potentially be derived from a range of hamstring-specific exercises that vary in the stimuli they provide. Therefore, a more rigorous approach is to select hamstring-specific exercises based on thoroughly constructed evidence-based frameworks surrounding the specific stimulus provided by the exercise, the accompanying adaptations elicited by the exercise, and the effects of these adaptations on hamstring functioning and injury risk mitigation when sprinting.

## Key Points

**Table:** 

An eccentrically lengthening, energy-absorbing, brake-driven model of hamstring function in the late-swing phase of sprinting has been widely supported across the scientific literature. However, it has recently been theorised that there is no eccentric muscle lengthening of the hamstrings during this phase.
Sprinting is a maximal-effort, high-velocity activity that exposes the hamstrings to large mechanical loads that are seemingly beyond isometric capacity. Accordingly, some active hamstring muscle fibre lengthening in the late-swing phase of the sprint cycle appears necessary to draw upon force enhancement mechanisms. This is anticipated to assist with decelerating the knee joint and tolerating the high mechanical loads experienced in the hamstrings.
Each individual hamstring MTU is expected to adopt various combinations of spring-, brake- and motor-driven functioning when sprinting, in accordance with their architectural arrangement and activation patterns.
Hamstring MTU functioning during sprinting is unique to this specific activity. Accordingly, hamstring-specific exercises for athletes required to sprint should not be selected on the basis of unvalidated claims of replicating hamstring function when sprinting. Rather, the selection of hamstring-specific exercises should be informed by thoroughly constructed evidence-based frameworks outlining the specific stimulus provided by the exercise, the accompanying adaptations elicited by the exercise, and the effects of these adaptations on hamstring functioning and injury risk mitigation while sprinting.

## Introduction

**Fig. 1:**
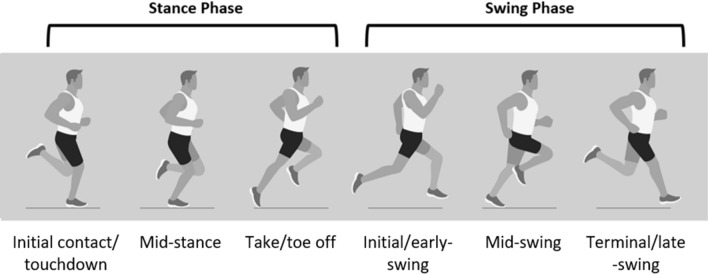
The phases of the sprint cycle [12]. Mid- and late-swing, where knee joint extension occurs at a high velocity prior to experiencing a rapid deceleration thereafter, expose the hamstring to high forces and are considered a key phase for the occurrence of hamstring injury

Muscles perform a variety of functions during locomotion, acting as motors, brakes, springs and struts [1]. The functioning of the hamstring muscles while sprinting is of particular interest to researchers and sporting practitioners alike, with this specific muscle group remaining a primary concern for athletic injury [2, 3], while also being a critical contributor to athletic performance capabilities [4, 5]. Interestingly, in opposition to the majority of existing research which posits an eccentrically lengthening, energy-absorbing, brake-driven model of hamstring function in the late-swing phase of sprinting (Fig. 1) [6–15], it has been theorised that there is no eccentric muscle lengthening of the hamstrings during this phase [16–18]. Specifically, the phenomenon proposed by Van Hooren and Bosch [16, 17] suggests that following passive lengthening of the hamstring musculature during the initial- and mid-swing phases of sprinting, the hamstring fascicles [interpreted as the contractile element (CE)] behave isometrically in the late-swing phase, immediately prior to ground contact [16]. It was further proposed that the movement presenting in late-swing is modulated by the series elastic element (SEE) (interpreted as the tendons and other connective tissues located outside of the muscle fascicles) “stretching and recoiling in a spring-like manner” [16]. This theory has been used as the primary justification for prescribing isometric hamstring exercises rather than eccentric hamstring exercises for athletes required to sprint [16, 17], a controversial notion that has gained substantial traction within the applied sporting world.

Due to current logistical challenges pertaining to the direct assessment of hamstring muscle–tendon behaviour while sprinting, there is presently a reliance upon models and theories to guide understandings of hamstring functioning during this specific activity. However, computational models exploring muscle–tendon function under dynamic conditions still utilise a number of assumptions and have many limitations. For example, Hill-type models typically assume uniform mechanical strain distributions along muscle fibres and tendons [15, 19], and they still do not accurately predict muscle forces under dynamic conditions [20–22]. These limitations ultimately restrict the development of precise understandings of hamstring muscle–tendon function when sprinting. As a result, theories surrounding hamstring function when sprinting continue to have widespread implications, influencing understandings of muscle–tendon behaviour and athletic injury mechanisms, as well as informing the development and implementation of athletic training, rehabilitation and injury risk mitigation protocols. While both the late-swing and early-stance/initial contact phases of sprinting are of interest in relation to hamstring injury [8, 9, 13, 23, 24], theories pertaining to hamstring function in the late-swing phase of sprinting are of particular importance, as this phase is widely regarded to be the primary stage of sprinting when hamstring injuries occur [7–9, 13, 14, 23, 25, 26]. Considering the widespread interest that exists across the sporting landscape relating to hamstring function when sprinting, the introduction of a new competing theory into the scientific literature, i.e. that there is no eccentric lengthening of the hamstring muscles in the late-swing phase [16, 17], evokes the need for the detailed exploration of this topic within the context of the available empirical evidence and contemporary muscle–tendon mechanics literature. Accordingly, the aims of this review article are three-fold: (1) to comprehensively explore the existing terminology, theories and models relating to muscle–tendon function and energetics during locomotion, (2) to relate these models to potential hamstring function when sprinting by exploring a variety of hamstring-specific research regarding muscle–tendon arrangement and architecture, electromyography (EMG), electromechanical delay (EMD), and lower-limb kinematics and hamstring kinetics when sprinting, and (3) to highlight the importance of developing and utilising evidence-based frameworks to guide hamstring training in athletes required to sprint.

## Clarifying the Terminology

### Muscle Contraction

Considering the importance that has been placed on contraction modality within the hamstring literature [7, 13–18, 27], a thorough exploration of the terminology surrounding muscle contraction and its various modalities is needed. This is necessary to both highlight the limitations of terminology commonly used to describe potential hamstring muscle function when sprinting, and for facilitating more sophisticated descriptions and understandings of hamstring muscle functioning in future research studies and academic discussions. There is widely accepted to be three main muscle contraction modalities: concentric, isometric and eccentric. These three terms, generally speaking, refer to active shortening, no change in length, and lengthening of the musculature, respectively. This inevitably results in a contradiction when describing a contraction (typically defined in dictionaries as “shortening” or “becoming smaller”) as either “isometric” or “eccentric”. However, due to the widespread usage of the term “contraction” within the muscle physiology vernacular, a simple (and most reasonable) proposed solution was to redefine the term in this context to mean “The active state of muscle; the attempt of muscle to shorten” with “no directionality to be inferred.” [28]. It is important to note that, due to the effects of activation and relaxation electromechanical delays (Table 1), electrical signalling and the generation of muscle tensions are not always occurring concurrently [29, 30]. Therefore, in the context of exploring hamstring function when sprinting, it is proposed that “active” be interpreted as generating active muscle tensions and not merely the presence of electrical signalling, as reasonably suggested elsewhere [6, 15, 16, 31, 32].

**Table 1:** Relevant nomenclature

Operational definitions	
Muscle contraction	The active state of muscle; the attempt of sarcomeres to shorten – no directionality to be inferred
Concentric contraction	“Shortening under (active) tension.” Commonly used at both macroscopic and microscopic levels of the musculature
Isometric contraction	“No change in length under (active) tension.” Commonly used at both macroscopic and microscopic levels of the musculature
Eccentric contraction	“Lengthening under (active) tension.” Commonly used at both macroscopic and microscopic levels of the musculature
Muscle fascicle	A bundle of muscle fibres that are surrounded by a connective tissue sheath called perimysium
Muscle fibre	Often described as a single muscle cell that contains many myofibrils, a skeletal muscle fibre contains many nuclei and is actually a syncytium formed by the fusion of many single-celled myotubes during development
Myofibril	A cylindrical organelle that runs longitudinally through a muscle fibre. It is composed of repeating units called sarcomeres
Sarcomere	The basic unit of muscle contraction. Sarcomeres incorporate the actin and myosin filaments, as well as many other proteins, e.g. filaments (titin, desmin, nebulin, etc.), structural proteins of the Z line and M band, etc.
Muscle–tendon unit (MTU)	A multifarious bodily structure primarily consisting of muscle and tendon and the connections between them. The mechanical behaviour of the MTU is commonly explored using Hill's three-element muscle model, which includes a contractile element (CE), series elastic element (SEE) and parallel elastic element (PEE)
Three-element Hill muscle model	A three-element representation of the mechanical responses of muscle conceived from AV Hill’s force–velocity relationship derived from experiments using tetanised muscle contractions [67], and further developed by Felix Zajac [68]. The model includes a contractile element (CE) and two non-linear spring elements, one in-series (SEE) and another in-parallel (PEE)
Contractile element/component (CE/CC)	Represents the active characteristics of the muscle fibres and is responsible for active force production which is generated by the actin and myosin cross-bridges at the sarcomere level
Series elastic element/component (SEE/SEC/SE)	Represents all elastic components of the MTU (i.e. the tendon, intramuscular filaments and cross-bridge elasticity) that are in-series with the contractile element
Parallel elastic element/component (PEE/PEC/PE)	Represents the passive force of the elastic elements that are considered in-parallel to the CE. Commonly simplified to refer to the connective tissues that surround the muscle fibres and fibre bundles, i.e. fascia, epimysium, perimysium and endomysium
Stretch–shorten cycle (SSC)	A basic muscle function presenting when a pre-activated muscle is stretched (eccentric action) prior to shortening (concentric) action. Notably, when a muscle fibre is activated, stretched, then immediately shortened, the force and power generated during the concentric action are greater than a concentric-only contraction [91]
Stress	Stress is defined as force per unit area and develops within a structure/tissue in response to an applied force. Stress is descriptive of the internal forces neighbouring particles of a given material exert on one another. Stress may be characterised as normal (force perpendicular to a plane) or shear (force parallel to a plane). Normal stress may be tensile or compressive depending on the mode of loading
Strain	Refers to the amount of deformation expressed as a normalised change in shape or size. Two basic types of strain exist: normal strain, which is related to change in length, and shear strain, which is related to change in angle. Normal strain is the ratio of deformation (lengthening or shortening) to original length and as such may be tensile or compressive. Shear strain is the amount of angular deformation that occurs in a structure. For example, a rectangle drawn on one face of a solid before a shear stress is applied will appear as a parallelogram during the application of a shear stress
Mechanical work	Mechanical work refers to the amount of energy transferred by a force and can be calculated as the product of the applied force and the displacement of the structure in the direction of the force. Mechanical work can be either negative (energy absorption) or positive (energy generation). There is a noteworthy inconsistency in muscle research in that a muscle fibre contracting isometrically does no work, yet the generation of muscle force clearly requires the expenditure of metabolic energy
Force enhancement	A phenomenon whereby muscle fibre force is enhanced above isometric levels through active (eccentric) stretch. Demonstrated by Katz [63] over 80 years ago
Residual force enhancement	The increase in isometric steady-state force following an eccentric contraction compared with the isometric steady-state force obtained during a purely isometric contraction at the corresponding length and activation [84]
Isometric fixed-end tetani	Refers to a specific type of muscle fibre contraction whereby the ends of the muscle fibre are fixed to an immovable resistance. This contraction is therefore characterised by a constant muscle fibre length
Creep	In muscle mechanics, the term creep can be used to describe two different scenarios: (1) tension creep, which refers to the gradual rise in tension that occurs during isometric fixed-end tetani. This rise in tension is commonly understood to be caused by the gradual deformation of sarcomeres and various viscoelastic components located within the muscle fibres during contraction. (2) Creep is also used to describe the gradual elongation of a muscle fibre (or other biological material) under a constant load
Spring-driven locomotion	A locomotive model of MTU functioning whereby muscles primarily act as mechanical struts, performing minimal work while the tendons perform the majority of work by storing and releasing elastic energy
Motor-driven locomotion	A locomotive model of MTU functioning that is more reliant upon muscle actuators and active muscle work. In this mode of functioning muscle fibres undergo relatively large excursions, shortening to produce power by performing mechanical work
Physiological cross-sectional area (PCSA)	Refers to the area of the cross-section of a muscle perpendicular to its fibres. A muscle's PCSA is representative of the maximal number of actin–myosin cross-bridges that can be activated in-parallel during contraction, and accordingly, the maximal force-generating capacity of a given muscle is largely proportional to its total PCSA [81, 92]. A muscle’s PCSA is generally measured at the largest point of the muscle
Pennation angle	Refers to the angle between a muscle fascicle or fibre orientation and the tendon axis. The pennation angle impacts the magnitude of the component of fibre force oriented along the muscle’s line of action that contributes to whole-muscle force [93, 94]
Variable muscle gearing	Refers to the rotation of muscle fascicles and fibres in pennate muscles during contraction. Variable muscle gearing provides pennate muscles with an “automatic transmission system” [93] that gears a muscle more favourably towards heightened shortening velocities or preserved force outputs depending on the mechanical demands of the required contraction [93]. Variable muscle gearing is commonly described by a gear ratio comparing muscle contraction velocity with muscle fibre velocity (muscle velocity/fibre velocity)
Activation electromechanical delay (A-EMD)	Time period between the onset of the electromyographic (EMG) signal and the onset of force production during a muscle contraction. The majority of A-EMD is attributed to the time required to take up MTU slack [95]. A-EMD is commonly reported to be between 30 ms and 100 ms in duration in humans
Relaxation electromechanical delay (R-EMD)	Time from the offset of muscle electrical activity to the offset of muscle tension. This delay is the result of electrochemical and mechanical processes. Specifically, the time taken for the reuptake of calcium into the sarcoplasmic reticulum, the detachment of actin–myosin cross-bridges, and SEE relaxation [96]. R-EMD is reported to be longer than A-EMD in humans and is commonly in excess of 200 ms [30, 96–99], reaching delay times as long as 366 ms [96]
Muscle slack	The delay between the start of CE activation and SEE recoil [100]. The removal of muscle slack has been purported to include alignment of the MTU, the uptake of slack in the aligned CE and SEE, changes in three-dimensional muscle shape, and SEE compliance [16, 100]

Within the scientific literature, terminology surrounding muscle contraction has been used in a variety of contexts to describe the movements of various structures, such as bony attachments or whole musculotendinous units (MTU), whole muscle, muscle fascicles and fibres, and sarcomeres. This diverse usage across both macroscopic and microscopic levels has created confusion as to what muscle contraction-related terminology actually refers to. For example, in the applied sporting world it is common to define or infer muscle contraction modality by the movement of bony attachments, which is considered to be reflective of the whole MTU. However, such descriptions are flawed as it has been demonstrated that neither the muscle nor the muscle fascicles or fibres behave isometrically during “isometric” MTU behaviour [33, 34]. For this reason, definitions specifically centred on the behaviour of the musculature and not the bony attachments or MTU as a whole provide more robust and favourable definitions of muscle contraction, but additional complexities exist. For example, muscle fascicle and fibre strains can be disassociated from the whole muscle [35]. While it is justifiable to describe both whole muscle and muscle fascicles or fibres using currently available muscle contraction terminology, it is the muscle fibres (and more specifically sarcomeres) located within the muscle that drive contraction. It follows that these structures are responsible for a range of phenomena relevant to locomotion and muscle function such as active force generation and eccentrically induced force enhancement (during active muscle fibre stretch) [36]. Due to their particular importance and relevance, muscle fascicle and fibre behaviour will be repeatedly referred to within this article. However, it is important to note that these are different muscular structures. While a muscle fascicle constitutes a bundle of muscle fibres, and therefore muscle fascicle behaviour is often used as a surrogate indicator of muscle fibre behaviour within the scientific literature, the organisation of muscle fibres within fascicles can vary substantially [37, 38]. There are a variety of intrafascicular muscle fibre arrangements that exist [37, 38], and it is common for individual muscle fibres to run for only a portion of a muscle fascicle, terminating intrafascicularly [37, 38]. Accordingly, inferences regarding muscle fibre behaviour from muscle fascicle behaviour should be made with caution.

### Do Isometric Contractions Exist?

Despite the widespread adoption and utilisation of the term “isometric” within the hamstring and broader sports science literature, the existence of this particular mode of contraction has been questioned [39]. When referring to isometric muscle contractions, Rodgers and Cavanagh [39] claim that “this condition probably does not exist”. While such a statement may be surprising considering the common use of the term, there are a number of reasons why isometric contractions would be extremely rare (and may not even exist), especially under dynamic conditions. Firstly, and as already alluded to, when a joint or MTU is fixed, typically the contractile components of a muscle shorten at the expense of the elastic structures in-series, i.e. the elastic structures stretch [31, 33]. At the macroscopic level, muscle fibres can shorten up to 28% during whole MTU “isometric” contractions [34]. It is for such reasons that Rodgers and Cavanagh [39] have questioned the existence of this particular mode of contraction. Secondly, if a strict fibre- or fascicle-focused definition is adopted, an isometric contraction arguably requires the external force experienced by each active muscle fibre within a given muscle (which may consist of different fascicular regions with varying fascicle and fibre orientations) [40] to be exactly equal to the forces generated by those fibres. This appears to be an unlikely phenomenon to occur outside of in vitro muscle fibre clamping. Indeed, it should be considered that architectural profiles (such as region-specific fascicle lengths, arrangements and orientations) [40, 41] and load distributions commonly vary within single muscles [42–44], as is observed in the hamstrings [40–42, 45]. As expected, then, non-uniform strains have presented within and across muscle fibres and fascicles located in different regions of a given muscle [43–47]. Intramuscular variations in mechanical behaviour are an important consideration for both the development of intricate understandings of hamstring muscle function when sprinting, and also for understanding hamstring injury mechanisms. Certainly, one modelling study has reported variable muscle tissue strains across the biceps femoris long head during high-speed running, in accordance with the structural dimensions of this particular muscle [45]. In addition, the non-uniformity of muscle strain may explain why the hamstrings exhibit site-specific variations in eccentrically induced muscle damage after extended running performance [48], with the distal and middle regions of the hamstring muscles presenting with significantly more muscle damage than the proximal regions [48].

### Shortcomings of the Terms Concentric, Isometric and Eccentric as Descriptors of Hamstring Muscle Function

**Fig. 2:**
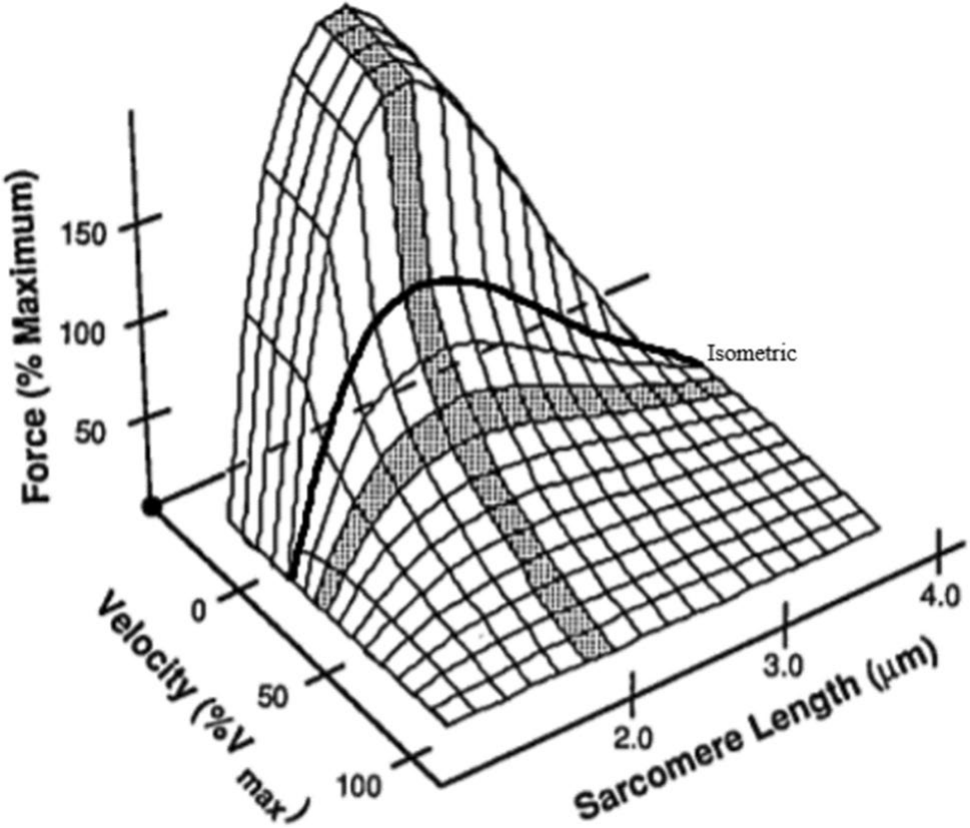
Three-dimensional depiction of a generalised relationship between sarcomere length, force and velocity within a single sarcomere. A similar relationship profile presents at both the muscle fibre and whole muscle levels. Adapted from Fridèn and Lieber [49] with permission

While common uses of the term “isometric” remain contentious, the familiar terms of concentric, isometric and eccentric all have major shortcomings when describing both hamstring muscle function when sprinting, and muscle function in general. These terms simply provide a generalised macroscopic description of the endpoints of the structure (e.g. MTU, muscle, muscle fibre, etc.) being referred to, the relevance of which remains uncertain in many contexts. Referring to their superficial nature, at the muscle fibre level, the three contraction modalities can occur at any conceivable muscle fibre length and contraction velocity (where applicable) (Fig. 2) [49]. At the whole muscle level, these contraction types can also occur under a wide spectrum of muscle tensions, the magnitude of which is dependent upon the neuromechanical regulation of muscle fibre engagement across the muscle. In addition, when applied at the muscle fibre level, these terms also do not clearly distinguish between any of the relevant internal biological mechanisms that drive muscle fibre functioning and force generation. To elaborate, sarcomeres behave non-uniformly during contraction (i.e. some sarcomeres can act eccentrically, while others act isometrically or concentrically), as has been exhibited during isometric fixed-end tetani [50–54]. Muscle fibres and sarcomeres also consist of a plethora of biological materials and structures (e.g. active and passive filaments, proteins of the Z line and M band, connective tissues, etc.) and irrespective of contraction modality, all biological material within a muscle fibre that experiences tension during contraction will inevitably be subject to some degree of strain, i.e. they deform/act eccentrically, as described by Young’s modulus. It is for this reason that the concerns raised by Rodgers and Cavanagh [39] regarding isometric contractions cannot simply be restricted to the macroscopic view of the MTU, but also remain relevant when describing the internal workings of the muscle fibres themselves. The gradual deformation of sarcomeres and various viscoelastic components located within muscle fibres during contraction is a feature of muscle fibre function that is understood to explain the increase in tension over time that is observed during isometric fixed-end tetani, a phenomenon termed “creep” [51, 55]. This raises questions regarding what specific structures are actually acting “isometrically” during an “isometric” contraction, besides perhaps the endpoints of a particular fibre, the mechanical relevance of which is often unclear in various contexts. Regardless, while the terms concentric, isometric and eccentric may, in some capacity, be linked to various muscular phenomena, it is important to acknowledge that these terms are descriptively limited, and muscle function can vary dramatically under any of these conditions. Indeed, it is the variably acting internal structures of the muscle fibre, such as sarcomeres and their accompanying active and passive filaments, that play fundamental roles in muscle functioning. Certainly, the internal structures of muscle fibres provide them with their internal viscoelastic properties [36, 56–61], and are also mechanistically responsible for a range of important muscular phenomena that are relevant to hamstring functioning when sprinting, such as force generation [36, 58, 59, 62] and force enhancement [36, 63] (Table 1).

**Fig. 3:**
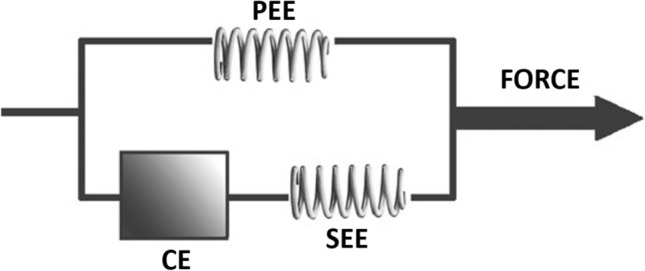
A three-element representation of the mechanical behaviour of muscle conceived from AV Hill’s force–velocity relationship derived from experiments using tetanised muscle contractions [67], and further developed by Felix Zajac [68]. Adapted from Wilson and Flanagan [69] with permission. CE, contractile element; SEE, series elastic element; PEE, parallel elastic element. See Table 1 for further explanations of the various components

The information in this section has not been presented to suggest that the macroscopic terms of eccentric, isometric and concentric should be abandoned. These terms are firmly entrenched in science, and they still provide general descriptive benefits that can be linked, in some capacity, to various muscular phenomena. Rather, the extensive nature of this section is provided to highlight two main points. Firstly, while it remains uncertain as to where exactly specific structures within the muscle fibre fit within the context of Hill’s three-element muscle model (Fig. 3) [64], it is clear that characterising the CE as the muscle fascicles and the SEE and parallel elastic element (PEE) as the connective tissue located outside of the muscle fascicles is an uncomfortable simplification with major shortcomings. Indeed, Hill himself cautioned against simplistic anatomical interpretations such as this, as these components were derived from mechanical experiments and not histological observation [65, 66]. In addition, Hill also acknowledged that elasticity (series and non-series) may also reside in the muscle fibres themselves [65, 67], which is now well documented [36, 56–60]. It follows that the outdated approach of treating the muscle fascicles as the CE with no elastic characteristics has resulted in the widespread disregard of the viscoelastic properties of muscle. Secondly, and most importantly, it is essential to emphasise that muscle function is complex, and current applications of the terms eccentric, isometric and concentric are vague and risk facilitating the adoption of caricatures of muscle function for utilisation in the applied sporting world. Indeed, muscle functioning can vary remarkably under any of the three contraction modalities, and the internal components of muscle fibres can behave in an almost limitless number of ways facilitated by the near infinite array of force–length and force–velocity combinations that exist at various levels within the musculature (Fig. 2). These are important details to consider when specificity of hamstring muscle function is being contemplated, especially when claims of functional replication when sprinting are used as the primary justification for the selection of certain hamstring-specific exercises over others based on perceived similarities in contraction modality.

### Further Terminology Concerns

**Fig. 4:**
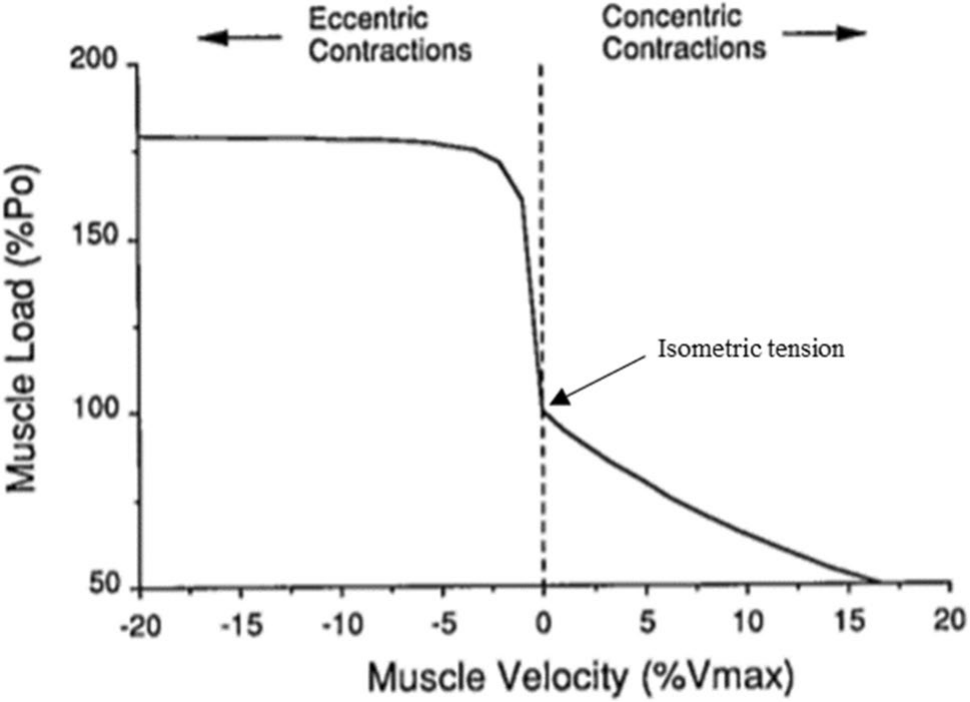
The classic force–velocity relationship. Po, isometric tension; %Vmax, percentage of maximal shortening velocity. Adapted from Fridèn and Lieber [49] with permission

In more recent years, more imprecise terminology such as “relatively-” or “quasi-isometric” has entered the literature to describe scenarios where relatively small muscle fascicle or fibre stretches, shortening or stretch–shorten cycles occur. This terminology is a source of confusion. Most scenarios adopting this language are in fact not isometric contractions, and labelling them as such has resulted in the neglect of the viscoelastic properties and particular functioning of muscle. For example, muscle spindles, which play a key role in the stretch reflex and the stretch–shorten cycle [70], are sensitive to submillimetre stretches [71, 72], while relatively small muscle fibre stretches (< 1 mm) also result in increases in muscle fibre tension [73]. These rises in tension occur at both fast and slow lengthening velocities [63, 73], as is depicted within the steeply rising, lengthening portion of the force–velocity curve (Figs. 2 and 4), and are termed “force enhancement” (Table 1). This is not trivial. Force enhancement provides a range of benefits to MTU functioning, such as heightened forces [63] at a relatively lower metabolic energy cost [74, 75], and a reduction in the number of active fibres required for a given force output [76–80]. These heightened forces also have implications for enhancing the amount of potential energy that can be stored in both the tendon and MTU as a whole [80, 81], which is relevant to muscles for which the primary role is to produce force with minimal length changes, as well as for muscles that perform work by shortening [82, 83]. Of additional terminological importance, while a muscle fibre may theoretically hold a fixed length after eccentric lengthening, it must be acknowledged that this is a different condition to a muscle fibre being activated at this same length, i.e. purely isometric contraction. Indeed, due to the prior eccentric lengthening, this muscle fibre will be in an eccentrically induced force-enhanced state [36]. Similarly, if a muscle fibre is actively lengthened prior to returning to its original length, the muscle fibre will continue to experience elevated tensions above those exhibited during a purely isometric contraction at that same original length, a phenomenon termed “residual force enhancement” (Table 1) [84]. If a detailed understanding of hamstring muscle functioning when sprinting is to be achieved, it is important that the particular workings of muscle are appropriately acknowledged and integrated into applied models and descriptions of muscle function.

While current applications of macroscopic terminology surrounding muscle contraction may offer some practical utility, they have also facilitated the integration of imprecise descriptions of muscle function into the scientific literature and applied sporting world. Unfortunately, this has both convoluted the theoretical foundations of our field and caused confusion. Despite this, further fixation on the nuances of terminology in this article may be a disservice, as to do so would arguably decontextualise and misrepresent some of the primary arguments regarding the intriguing spring-like hamstring phenomenon that has been proposed by Van Hooren and Bosch [16, 17]. Indeed, across the animal and human scientific literature investigating locomotion, spring-driven MTU functioning utilising low levels of muscle work is relatively common [85–90]. Accordingly, prior to examining the existing hamstring-specific literature, a detailed examination of the various models of MTU functioning during locomotion (and some of their underlying assumptions) that may be applicable to hamstring function while sprinting is needed. This will help with contextualising and assessing the relevance and applicability of the spring-like isometric hamstring model of functioning that has been proposed by Van Hooren and Bosch [16, 17].

## Models of Locomotion

To better contextualise and clarify the relevant theories, models and propositions that may be applicable to hamstring functioning when sprinting, it is important to understand the existing research relating to MTU behaviour during locomotion. Indeed, considering the recent calls to replace the eccentrically lengthening, brake-driven model of hamstring function in the late-swing phase of sprinting with an isometric spring-driven model [16, 17], exploration of the theoretical underpinnings surrounding relevant models of MTU functioning during locomotion, some of their underlying assumptions, and the contexts within which these models of MTU functioning have been proposed and observed is needed. This will better highlight their potential applicability to hamstring functioning across the entirety of the sprint cycle.

Two main models of MTU functioning during locomotion have been outlined in the literature: an efficiency model and a power model [80, 101]. The efficiency model, which is proposed for prioritising metabolic efficiency during locomotion, predominantly centres on spring-driven MTU behaviour. In this mode of functioning, muscles primarily act as mechanical struts, performing minimal work while the tendons perform the majority of work by storing and releasing elastic energy [80]. Spring-driven MTU functioning is widely considered to improve locomotor efficiency [81, 90, 102–105], and is an MTU behaviour that is commonly observable across a range of animal species [85–87], including humans [88–90]. For sustained high-velocity or high-intensity efforts, whereby the need to conserve energy becomes secondary to the immediate need for heightened power outputs, a motor-driven power model of MTU functioning that is more reliant upon muscle actuators and active muscle work has been theorised [80, 101]. Specifically, during high-intensity locomotive conditions, it is expected that muscle fibres commonly undergo relatively large excursions, with the muscle fibres shortening to produce power by performing mechanical work [80, 101]. Interestingly, in circumstances where extremely large power outputs beyond the capabilities of contracting muscle are required, such as during a mantis shrimp strike, a specialised spring–latch arrangement can be observed [106, 107]. However, such a mechanism is largely unique to crustaceans and is markedly different to mammalian utilisation of elastic tissues such as tendons.

The function and predisposition of a given MTU to operate in a particular manner can largely be anticipated from its architectural arrangement [80]. For example, muscles with relatively long muscle fibres have greater working ranges [108] and are favourably designed to produce movement, generate mechanical power, and absorb energy by either shortening or lengthening with relatively large amplitude displacements [109]. In contrast, muscles with short, pennate fibres and a large physiological cross-sectional area (PCSA) (and therefore a large quantity of biological material in-parallel), are better suited to generate force with limited length changes [80, 109]. These muscles are often accompanied by long, compliant tendons, with the high forces and limited muscular length changes permitting elastic strain energy to be stored and recovered within the tendon [80]. Such a design is perhaps best exemplified by the MTUs of certain common animals, such as horses, camels and kangaroos [110, 111]. In humans, the closest example of such a design is arguably the triceps surae.

### Spring-Driven Locomotion: Would Isometric MTU Work Loops Offer Metabolic Savings for the Hamstrings when Sprinting?

For submaximal locomotive conditions (such as walking and hopping), a spring-driven model of MTU functioning primarily relying on tendon work contributions is commonly utilised [89, 90]. Within this particular model, it has widely been assumed that isometric MTU work loops, i.e. where the tendon performs all of the mechanical work by storing and releasing elastic energy while the muscle fibres generate force isometrically, is the most efficient manner to operate. Specifically, this behaviour has generally been considered to reduce the metabolic energy cost of locomotion by restricting muscle work [16, 81, 103, 105, 110–113]. Accordingly, one potential argument for the prioritisation of an isometric spring-driven model of hamstring function when sprinting may revolve around metabolic energy savings over the course of the sprint cycle. The conclusion that minimising muscle work offers metabolic savings is rather intuitive as muscles consume significant metabolic energy when required to generate mechanical energy (when shortening) [74, 114, 115], while tendons do not consume metabolic energy in their role as springs due to their passive elastic nature. In addition, tendons exhibit a heightened capacity for energy return (tendons commonly return approximately 93% of the stored energy) [116], while musculature, although highly effective at energy absorption, is typically understood to have a reduced capacity for energy return [81]. When considering these characteristics of muscle and tendon, it appears logical that prioritising tendon stretch and recoil over muscle work by having the hamstrings contract isometrically would save metabolic energy when sprinting. However, this assumption requires deeper critical evaluation, as outlined herein.

**Fig. 5:**
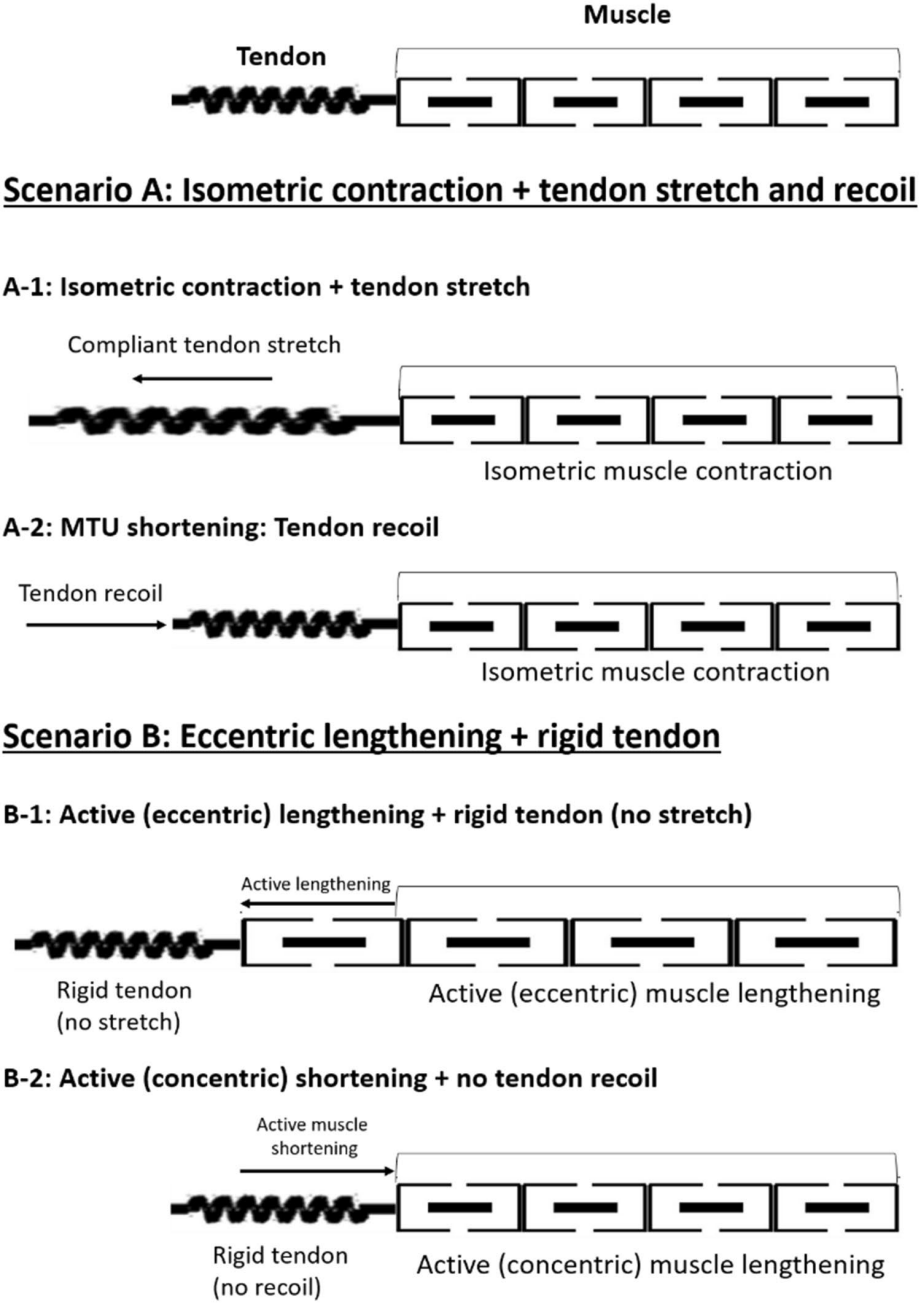
A diagram displaying two scenarios explored by Holt et al. [114]. In scenario A, a compliant tendon allows the muscle to operate isometrically as the tendon stretches to store mechanical energy (**A-1**), and then recoils to return it (**A-2**). In scenario B, the tendon is more rigid and all of the cyclic work must be performed by the muscle; whereby the muscle actively lengthens to absorb mechanical energy (**B-1**) prior to shortening to produce it (**B-2**)

Until 2014, the long-held assumption that isometric work loops offer metabolic energy savings during locomotion compared to muscular stretch-shorten cycles had never been tested. Notably, Holt et al. [114] directly investigated the energetic cost of force production during isometric cycles and muscular stretch–shorten cycles (Fig. 5), demonstrating no detectable differences in the metabolic cost of force production between them. This is a most intriguing finding that challenges both the widely held assumption that isometric work loops offer metabolic savings during locomotion compared with muscular stretch–shorten cycles, as well as the assumption that reductions in muscle work drove the evolution of long, compliant tendons in the distal limbs of cursorial species [114]. While this evidence may be surprising, there are logical explanations. At the forefront, tendons do not function independently of muscles; rather, they act in-series. To stretch and gain potential energy, a tendon requires an opposing force from the muscle [117]. Therefore, to store elastic energy in the tendon the attached muscle is still required to produce force at some metabolic cost [114]. This is an important concept, and a major limitation of work-based approaches to analysing locomotor energetics is that they typically do not take into account the energy consumed by muscles that primarily produce force but not work, such as occurs when a muscle operates “isometrically” or is lengthened when active. While it is clear from existing understandings of the energetics of muscle function that the energetic cost of force production during muscle shortening should be higher than that of isometric force production [67, 74, 115, 118], when muscle is lengthened while active, it will produce heightened forces [63] at a relatively lower metabolic energy cost [74, 75]. It follows that, while the energy cost of active muscle shortening (Fig. 5B-2) may be higher than isometric contractions (Fig. 5A-1 and A-2), the energy cost of force production during the lengthening portion of a muscular stretch shorten cycle (Fig. 5B-1) will be lower than isometric contractions (Fig. 5A-1 and A-2). The net result is that that there are no detectable differences in the metabolic cost of force production between muscular stretch–shorten cycles and isometric contractions [114]. Most importantly, if the assumption that isometric MTU work loops conserve metabolic energy during locomotion is accurate, and this behaviour in the hamstrings would be energetically beneficial when sprinting, the metabolic cost of muscle force production should be higher during muscular stretch–shorten cycles compared with isometric contractions, which is not the case [114].

Holt et al. [114] investigated a widespread and long-held assumption regarding locomotive energetics, presenting some thought-provoking results in the process. Indeed, it was proposed that the energetic benefits of short muscle fibres drove the evolution of long, compliant tendons in the distal limbs of cursorial species (including humans) rather than reductions in muscle work [114]. These benefits include (1) concentrating muscle mass proximally within the limb, reducing inertia and swing costs [119–122], and (2) decreasing the cost of force generation through reductions in muscle volume to cross-sectional area ratio [123]. It was further concluded that the absorption and production of mechanical energy is likely to be distributed between muscle and tendon during locomotion, with long compliant tendons (such as those observed in the triceps surae and hamstrings of humans) reducing rather than eliminating muscle work [114]. Interestingly, this conclusion is in alignment with those initially proposed by Biewener [80], who also theorised that, for MTUs where the primary role of the muscle is to generate force, it was common for muscles to actively lengthen, with the accompanying force enhancement a likely contributor to improved elastic energy savings within a long tendon. Similarly, Lindstedt et al. [60] provided sound evidence that there is an active muscle spring that serves to store and recover elastic strain energy during locomotion, which also stiffens in response to locomotive training. Reasons such as these likely explain why “near isometric” muscle behaviour is not ubiquitous in animals reliant upon spring-driven locomotion and that exhibit long, compliant tendons [124–126]. For example, active muscle fibre lengthening and shortening have been recorded in both guinea fowl [124, 125] and goats [126] during locomotion. In humans, the triceps surae is arguably the muscle group that most closely reflects the short-fibred and long-tendoned MTU arrangements of many cursorial species reliant on spring-driven locomotion. Interestingly, small fascicle stretches have been observed in the triceps surae of humans during a variety of locomotive tasks requiring spring-driven function from this particular muscle group, such as walking [88, 89], running [70, 89], sprinting [127] and submaximal hopping [90, 128]. Despite these findings, further investigations in this area are needed as within the constraints of a given in vivo system of MTU functioning during locomotion, the precise distributions of work between muscle and tendon, and the specific combinations and timings of particular muscle and tendon behaviours, may yet have important implications for both energetics and power outputs [80, 81, 101, 129–132]. The exact nature of these relationships may be integral for developing more thorough understandings of hamstring functioning when sprinting.

### Motor-Driven Locomotion and Active Muscle Lengthening: Is It Applicable to the Hamstrings When Sprinting?

While spring-driven MTU functioning that has a strong reliance upon tendon work is commonly observable in humans and the wider animal kingdom, locomotive strategies that are more strongly reliant upon muscle work are also apparent. Indeed, Biewener [80] theorised that during ballistic and high-intensity locomotive efforts, whereby the need to conserve energy becomes secondary to the immediate need to maximise power outputs, a motor-driven model of functioning dependent upon muscle fibre work may be adopted. It was specifically theorised that under these conditions the muscle fibres are required to shorten to produce power by performing mechanical work. To support this theory, Biewener presented findings displaying considerable fibre shortening in the pectoralis of pigeons during the downstroke of flight [80]. In this scenario, the majority of fascicle lengthening during the upstroke was conducted under passive conditions. However, a small degree of active stretch of the muscle’s fibres was observed late in the upstroke, with this phenomenon anticipated to play a critical role in enhancing force development [80]. This is not the only example of locomotive strategies that are dependent upon muscle work in the animal kingdom, however. Some of the largest mammals on earth, such as the rhinoceros, elephant and blue whale, use a predominantly motor-driven system for propulsive activities [133, 134]. Interestingly, under such circumstances tendons do not serve to store and release elastic energy; rather they primarily act as transmitters of muscle contractile force to the origin-insertion points for generation of limb movements [101].

While obvious differences exist between pigeons, elephants and humans, the latter present with notably unique and multi-faceted MTUs that exhibit extraordinary diversity in function and adaptability [68]. Spring-driven and motor-driven modes of functioning are not necessarily exclusive of one another, and humans are indeed capable of using various combinations of spring- and motor-driven MTU behaviours depending on the demands of the specific task at hand [68, 101, 135, 136]. For example, while humans commonly adopt a spring-driven model of functioning of the triceps surae when hopping, during a maximal-effort countermovement hop, humans primarily adopt a motor-driven model of functioning reliant upon muscle actuators [101]. Indeed, despite the seemingly specialised spring design of the triceps surae, considerable eccentric and concentric muscle work contributions were exhibited during single maximal-effort hopping performance, with the tendons contributing only 35% of the total work [101]. This is a most intriguing finding that not only highlights the ability of humans to manipulate MTU behaviours depending on movement requirements, but also provides compelling evidence that muscle actuators, not springs, drive maximal-effort locomotion in humans [101].

Although spring- and motor-driven MTU functioning (and various combinations of these) are frequently observed in both humans and the wider animal kingdom, muscles are also commonly required to act eccentrically under both of these conditions. Specifically, when the force applied to a muscle exceeds the force produced by the muscle, the muscle will lengthen [137]. This phenomenon can occur at both high and low loads, with the neuromechanical regulation of tensions and stiffness permitting lengthening contractions at submaximal levels when needed [138, 139]. Eccentric muscle lengthening occurs when controlled muscle lengthening is a required component of a given movement solution and/or when a muscle is required to act as a damper or shock absorber to absorb mechanical energy and then dissipate the energy as heat [137–139]. Eccentric muscle lengthening does not only serve to absorb and dissipate energy, however. Through force enhancement mechanisms, active muscle stretches also serve to enhance force development [63], which can increase the potential energy stored in the tendon [80, 81], and minimise energy expenditure/unit of force generated [63, 74, 75, 80]. In addition, a portion of the energy absorbed in the muscle during locomotion can also be stored temporarily as elastic recoil potential energy within the muscle and subsequently recovered through the stretch–shorten cycle [60, 91]. This function is time dependent, meaning that, if the energy is not recovered, it is subsequently lost as heat [140]. When considering all of these characteristics of eccentric muscle functioning, it is evident that eccentric muscle behaviour is a valuable feature of muscle functioning that provides enhanced MTU functionality and control, and also bestows a useful means of regulating the dynamics of the tendon spring [36, 60, 81, 137].

Considering the widespread applications of eccentric muscle functioning, it is perhaps unsurprising that eccentric muscle behaviour can commonly be observed in humans in a variety of muscles and during a range of spring-driven and motor-driven locomotive activities requiring force production, energy absorption and/or utilisation of the stretch–shorten cycle. This includes but is not limited to loaded exercises involving controlled lengthening contractions of the hamstrings, such as the Nordic hamstring curl [141–143], single-leg Roman chair [141], and single-leg deadlift [141], loaded exercises involving lengthening contractions of the tibialis anterior [138], gastrocnemius and soleus [144–147], and the vastus lateralis and vastus intermedius muscles [148, 149], downhill running [150], eccentric cycling [151], rowing [152], and countermovement and drop jumps [153–155]. As already noted, small fascicle stretches have also been observed in the triceps surae during the spring-driven activities of walking [88, 89], running [70, 89], sprinting [127] and submaximal hopping [90, 128]. During certain locomotive tasks, the degree of fascicle stretching that presents also appears to share a relationship with intensity. For example, it has been demonstrated that the duration, amplitude and velocity of active fascicle stretches increase in the vastus lateralis with heightened rowing intensities [152]. Similarly, drop jumps from relatively high heights result in sudden stretches of the muscle fascicles located in the triceps surae [153, 154]. Perhaps most relevant, despite the spring-oriented architecture of the triceps surae, during maximal-effort locomotion the majority of work is performed by the muscle and not the tendon, with considerable eccentric and concentric muscle work contributions being observed during this particular hopping task [101].

When taking into account the intensity of sprinting, and that eccentric muscle behaviour is a common feature of both spring-driven and motor-driven muscle functioning, it appears likely that the hamstring muscles would adopt a model of functioning that has some reliance upon both muscle actuators and active muscle lengthening. However, despite providing enhanced MTU functionality, eccentric muscle lengthening is also highly damaging to a muscle [49, 156–158]. Indeed, it is active fibre strain, and not stress, that is primarily responsible for eccentrically induced muscle damage [49, 156, 157]. This is an important consideration for athletes required to sprint. The accumulation of eccentrically induced muscle damage has reasonably been suggested to be a potential causal factor for hamstring injury occurrence during high-speed running [6]. Accordingly, strategies that assist with reducing hamstring fibre strains when sprinting may have an important role in preserving the hamstring musculature by limiting the accumulation of muscle damage [156], minimising muscle damage-related losses in muscle force production, stiffness and function [159], and potentially reducing susceptibility to injury.

## Hamstring Muscle–Tendon Arrangement and Architecture: Insights into Hamstring Function When Sprinting

It is well documented that MTU arrangement (the manner in which muscles and tendons are organised relative to one another) and architecture (the physical properties and characteristics of the muscle and tendon tissues themselves) holds significant implications for muscle–tendon functioning [80, 81, 108]. A variety of MTU arrangements and architectures exist, and accordingly, a vast array of functional differences can be observed between the many MTUs presenting across the human body and wider animal kingdom [80, 81, 108]. Importantly, these characteristics can provide valuable insights into the particular functioning and roles of a given MTU [80, 81, 108], such as those making up the hamstrings. For example, parallel muscles, whereby the muscle fibres run parallel to the tendon, commonly present with relatively long fascicles and are therefore typically well suited to higher velocity contractions and large fascicular excursions [80, 81, 108]. In contrast, pennate muscles, whereby the muscle fascicles and fibres are arranged at an angle to the tendon, allow for a greater number of muscle fibres to be packed in parallel for a given muscle volume, increasing the force-generating capacity of the muscle [80, 81, 108]. However, these muscles typically exhibit shortened fascicles, restricting their fascicular displacements and velocities compared with longer-fibred parallel muscles [80, 81, 108]. These muscles are also commonly accompanied by long connective tissue and tendinous structures that contribute to the absorption and release of energy [80, 81, 108].

A number of architectural characteristics and measures exist, with each having a distinct influence on muscle functioning. For example, a muscle’s PCSA is representative of the maximal number of actin–myosin cross-bridges that can be activated in parallel during contraction, and accordingly, the maximal force-generating capacity of a given muscle is largely proportional to its total PCSA [81, 92]. Fascicle lengths have important implications for force–velocity [160, 161] and force–length relationships [162, 163], fibre and muscle shortening velocities [94], and the capacity of a fascicle to do work and tolerate strain [52, 164]. In pennate muscles, the pennation angle impacts the magnitude of the component of fibre force oriented along the muscle’s line of action that contributes to whole-muscle force [93, 94]. In addition, pennate muscles exhibit variable muscle gearing [35, 93, 94, 165] which provides dynamic alterations to the force, displacement and velocity characteristics of these muscles by rotating muscle fibres and adjusting pennation angles depending on the mechanical demands of a contraction [35, 93, 94, 165]. There are numerous architectural and mechanical characteristics that exist; however, due to their particular importance to muscle and MTU functioning, PCSA, fascicle length, pennation angle and tendon length will be the main focus of the following section, providing a general macro-level insight into the arrangement and architecture of the hamstring muscles.

### Hamstring Muscle–Tendon Architecture: A Comparison between Muscles

The hamstrings consist of three main muscles; semitendinosus, semimembranosus and biceps femoris, which has two heads: long head (BFlh) and short head (BFsh). Each individual hamstring MTU is unique, differing with respect to muscle fibre arrangement, PCSA, fascicle lengths, pennation angles, volume and connective tissue characteristics among other features (Table 2) [40, 42, 166]. In addition, some of these architectural characteristics, such as fascicular arrangement, fascicle lengths and pennation angles, display high variability within individual hamstring muscles [42]. Relative to one another, some of the hamstring muscles, such as semitendinosus and BFsh, are arranged in a manner that appears most beneficial for facilitating large fascicular excursions. Indeed, both semitendinosus and BFsh display relatively small PCSAs, long fascicles and low pennation angles, while BFsh also presents with a relatively short distal tendon. It follows that, semitendinosus has been appropriately described as a long, thin and straplike muscle, while BFsh has been described as having a thin muscle belly, that is broad and long [40]. In contrast, semimembranosus and BFlh have been described as pennated [166] and bipennate [40] in appearance, and display relatively large PCSAs, short fascicles, greater pennation angles and long distal tendons. Such an arrangement is suggestive that, relative to semitendinosus and BFsh, these muscles have an increased capacity for force generation [167] and spring-driven behaviour, and a reduced capacity for fascicular lengthening and shortening. Considering these characteristics, it is perhaps unsurprising that semimembranosus is understood to exhibit the highest peak forces when sprinting [14], followed by BFlh [14], which also experiences the largest peak strains (~ 12%) [6, 7, 14]. Notably, semitendinosus, semimembranosus and BFlh all exhibit prominent connective tissue structures, e.g. aponeuroses and distal tendons (Table 2) [40]. When considering these characteristics, it is anticipated that, when sprinting, there are substantial tendinous and connective tissue contributions to hamstring functioning, as has been stipulated by existing hamstring modelling studies [7, 14]. Specifically, these structures are expected to act as mechanical buffers that absorb energy during active MTU lengthening, protecting the muscle fibres from excess strain, and potentially acting as a spring that releases this energy for utilisation in the subsequent phases of the sprint cycle [7, 81].

**Table 2:** Comparison between selected architectural measures of the hamstrings and the triceps surae

	PCSA (cm^2^)	Fascicle length (cm)	Pennation angle (°)	Distal tendon length (cm)
Hamstrings				
Semitendinosus	8.1 [40]	9–24 [42]	0–18 [42]	25 [40]
Semimembranosus	15.8 [40]	5–8 [42]	15–31 [42]	26.1 [40]
Biceps femoris long head	10.1 [40]	5–14 [42]	0–28 [42]	27.5 [40]
Biceps femoris short head	3 [40]	10.4–14 [42]	10–16 [42]	11.2 [40]
Total	37 [40]			
Triceps surae				Achilles tendon length (cm)
Medial gastrocnemius	51 [168]	5.7 [168]	21.4–22 [169]	18.1–22.5 [170–172]
Lateral gastrocnemius	24 [168]	6.6 [168]	10.2–11 [169]	
Soleus	131 [168]	3.9 [168]	23.5–25.7 [169]	
Total	206 [168]			

### Hamstring Architecture and the Spring-Oriented Triceps Surae: An Architectural Comparison

Due to its distinctly spring-oriented design and functioning, the triceps surae provides an intriguing model for comparison with the hamstrings to help discern any potential similarities in function. As noted, the triceps surae exhibits a unique muscle–tendon structure, whereby the relatively long elastic connective tissue (tendon and aponeurosis) accounts for the majority of each MTU’s length, and is accompanied by muscles consisting of relatively large PCSAs (soleus; 131 cm^2^; medial gastrocnemius; 51 cm^2^; lateral gastrocnemius 24 cm^2^) and short muscle fibres (3–7 cm across the three triceps surae muscle groups) (Table 2) [168]. These characteristics present a design that is particularly well suited to generating force (which assists with resisting fascicles stretches during forceful interactions with the ground) and to store and release elastic energy in connective tissues during spring-driven locomotion. In contrast, the hamstrings display some notable architectural differences to the triceps surae. While both the gastrocnemius and soleus muscles are pennated, providing a shared characteristic with the hamstring muscles [42], soleus and the medial gastrocnemius exhibit a PCSA that is more than 8 and 3 times the size of semimembranosus (the hamstring muscle with the largest PCSA), and more than 13 and 5 times the size of BFlh, respectively. In total, the three triceps surae muscles display a PCSA of ~ 206 cm^2^ [168], which is more than five times larger than the ~ 37 cm^2^ observed for all of the hamstring muscles combined [40]. In addition, the hamstring muscles also consist of relatively long fascicles compared with the 3–7 cm observed in the triceps surae [168] (Table 2). It follows that the proportion of fascicle length to PCSA differs substantially between the hamstring muscles and the triceps surae, with the triceps surae being particularly well suited for force generation with minimal fascicular excursions, and the longer-fascicled and thinner hamstring muscles having a reduced force-generating capacity while also appearing to better accommodate larger fascicular excursions. These architectural differences are important to consider as the mechanical demands placed on these muscle groups also differ when sprinting. To elaborate, the triceps surae plays an important role in stabilising the ankle joint, particularly during foot ground contact. After toe down, the triceps surae resists ground contact forces and the ankle undertakes a relatively small degree of flexion (~ 20°) [12]. The heightened ankle joint and leg stiffness that presents when sprinting contributes to maximal sprint velocities by providing a stiff rebound when interacting with the ground [173]. This is in contrast to the considerably larger ~ 140° excursion experienced at the knee joint during the swing phase of sprinting [12], whereby opposed to managing interactions with the ground, the hamstrings are tasked with countering the rapid knee extension velocities (> 1000°/s) by stretching to maximum or near-maximum lengths in late-swing [8, 11, 15, 25],

### Variable Muscle Gearing: An Important Consideration for Pennate Hamstring Muscle Function When Sprinting

While pennated muscles allow for a greater number of muscle fibres to be packed in parallel, this arrangement also provides an additional functional feature that is unique to this particular muscle arrangement: variable muscle gearing [35, 93, 94, 165]. As the muscle fibres in pennate muscles are oriented at an angle to the muscle’s line of action, they can rotate during contraction, altering the pennation angles observed within the muscle. This rotation is determined by dynamic changes in muscle shape (muscle thickness and width), which is currently understood to be controlled by an interplay between contractile forces and connective tissue constraints [93, 165]. Notably, variable muscle gearing provides pennate muscles with an “automatic transmission system” [93] that gears a muscle more favourably towards heightened shortening velocities or preserved force outputs depending on the mechanical demands of the required contraction [93]. To elaborate, when a muscle shortens during low-force and high-velocity contractions, the connective tissues surrounding the muscle offer enough resistance to prevent increases in muscle width. Due to the isovolumetric nature of muscle, subsequent increases in muscle thickness occur [93]. This rising muscle thickness results in fibre rotation, which increases the pennation angles within the muscle during the contraction, elevating the contraction velocity of the muscle both in absolute terms and relative to the fibre contraction velocity [93]. During high-force contractions, the heightened forces experienced overcome the resistance to increases in muscle width provided by surrounding connective tissues, and the muscle increases in width. Muscle thickness subsequently decreases, limiting the rotation of muscle fibres during the contraction [93]. As muscle fibre rotation is restricted, pennation angles remain relatively low, which ensures that a larger component of the forces generated by the muscle fibres contribute to whole muscle force [93]. Due to its effects on muscle contraction velocity, variable muscle gearing is commonly described by a gear ratio comparing muscle contraction velocity to muscle fibre velocity (muscle velocity/fibre velocity) [93, 165]. A high muscle gear is reflective of a large degree of fibre rotation during contraction and therefore a heightened muscle shortening velocity relative to fibre shortening velocity. A low muscle gear is reflective of a relatively low degree of fibre rotation during contraction, and therefore a muscle shortening velocity that more closely aligns to that of the muscle fibres.

Interestingly, variable muscle gearing has similar effects during eccentric contractions [35]. It has been reported that pennate muscles function at a relatively high gear ratio when actively lengthening, resulting in larger stretches in the muscle relative to the fascicles or fibres [35]. Logically, the relatively heightened gear ratio exhibited during lengthening contractions exists to reduce the strains experienced by the fascicles or fibres when the muscle is lengthened, protecting the muscle fibres from excess amounts of strain and eccentrically-induced muscle damage [35]. As all of the hamstring muscles exhibit some degree of pennation [42], variable muscle gearing is likely an important consideration for understanding hamstring functioning when sprinting, reducing active fibre lengthening in late-swing to protect the hamstring muscle fibres from excess strains. Currently, it is unknown to what extent muscle gearing may restrict fascicle and fibre lengthening in the hamstrings during the late-swing phase of sprinting, nor is it known to what degree it influences hamstring functioning during the rest of the sprint cycle. However, despite the potential effects of muscle gearing in these muscles, active hamstring fascicle lengthening has been reported during a range of eccentric-focused hamstring exercises [141, 142].

### Summary

Muscle–tendon arrangement and architecture provide useful insights into potential MTU functioning during locomotion. Discrepancies in MTU arrangement and architecture are therefore beneficial for developing theory surrounding the specific roles and functions of the individual hamstring muscles when sprinting, and for guiding hamstring modelling studies [14]. However, human muscles exhibit extraordinary adaptability and are capable of using various combinations of motor-driven and spring-driven MTU behaviours depending on the demands of the specific task at hand [101, 135, 136], even in the particularly spring-oriented triceps surae [101]. Accordingly, further exploration of the hamstring-specific literature available is needed to better clarify their potential functioning when sprinting. For more detailed insights into hamstring MTU arrangement and architecture, the reader is directed to other published literature [40, 42].

## Muscle Slack, Muscle Activation and Electromechanical Delay in the Hamstrings

In the theory proposed by Van Hooren and Bosch [16–18], it was maintained that the hamstring muscles passively lengthen during the initial- and mid-swing phases of sprinting, a phenomenon permitted by the existence of muscle slack (Table 1), prior to contracting isometrically in late-swing [16–18]. Accordingly, this theory proposed that there is no eccentric hamstring lengthening while sprinting. Indeed, the proponents of this theory criticised currently available modelling studies, which have indicated a large reliance upon eccentric hamstring function when sprinting [6, 11, 14, 15], for overlooking the effects of muscle slack [16, 17]. It was contended that these models required a minimum amount of activation, negating the existence of muscle slack and leading to the misconception that the hamstrings are lengthening eccentrically during the swing phase [16]. While this is an intriguing suggestion, and some EMG and EMD research was presented in an attempt to support this assertion [16], this claim is difficult to substantiate from the available EMG and EMD research. Currently, EMG studies investigating the hamstrings when sprinting present inconsistent findings, with some showing that the hamstrings receive electrical signalling during the entirety of the sprint cycle [11, 174–176], and others demonstrating that they do not [6, 15, 177, 178]. While it is not immediately clear as to why the EMG literature presents such inconsistent findings, some potential explanations may include measurement artefact due to various issues such as discrepancies in equipment quality or crosstalk [179]. Additionally, as a result of the variability in EMD [both activation-EMD (A-EMD) and relaxation-EMD (R-EMD); Table 1] that exists across the literature [180–185], there is also a large amount of uncertainty when attempting to infer the onset and offset of muscle tensions from EMG data [29]. To exemplify this, A-EMD is commonly observed to be between ~ 30 ms and 100 ms and has been reported to be as low as 24 ms in the hamstrings during eccentric contractions [184, 185] and as high as 127 ms in the hamstrings during isometric contractions [180–183], providing a large range of A-EMD possibilities. Further, R-EMD during voluntary contractions in humans is notably long (knee extensors: 200–350 ms [30, 97, 98]; plantar flexors: ~ 283.4 ms [99]; tibialis anterior: ~ 312 ms [97]; biceps brachii: ~ 366 ms [96]; no knee flexor data reported). Considering that for sprinters the sprint cycle typically lasts between 414 ms and 452 ms [186], R-EMD is an important consideration as time constraints may prevent tensions from completely dissipating during any periods of electrical signalling absence, averting the reintroduction of slack (the main contributor to A-EMD) [95] back into the MTU.

Despite the above-mentioned uncertainties, it is worthwhile exploring whether any reasonable hypotheses may be formed regarding hamstring engagement when sprinting based on the available EMG and EMD research. For example, considering muscle tensions present at low levels of muscle activation (below 5% of maximum EMG) and rise with increased electrical signalling [187], if the hamstring muscles are receiving some magnitude of electrical signalling for the entire sprint cycle, it appears reasonable to assume that the hamstrings would be under a constant state of tension and muscle slack would not be present. Additionally, considering the large knee joint ranges and MTU length changes that occur when sprinting [6, 7, 12, 15, 25], it is also reasonable to hypothesise that the hamstring fascicles would be required to actively (eccentrically) lengthen and shorten to complete the sprint cycle. Otherwise, for hamstring lengthening to be entirely absent, there would also have to be no shortening, and the constantly active hamstring muscles would be required to behave isometrically for the entire sprint cycle, which seems implausible. However, if the hamstrings are only receiving electrical signalling for a portion of the gait cycle, as reported in multiple scientific studies [6, 15, 177, 178], forming conclusions regarding the nature and timings of hamstring engagement becomes considerably more challenging.

**Fig. 6:**
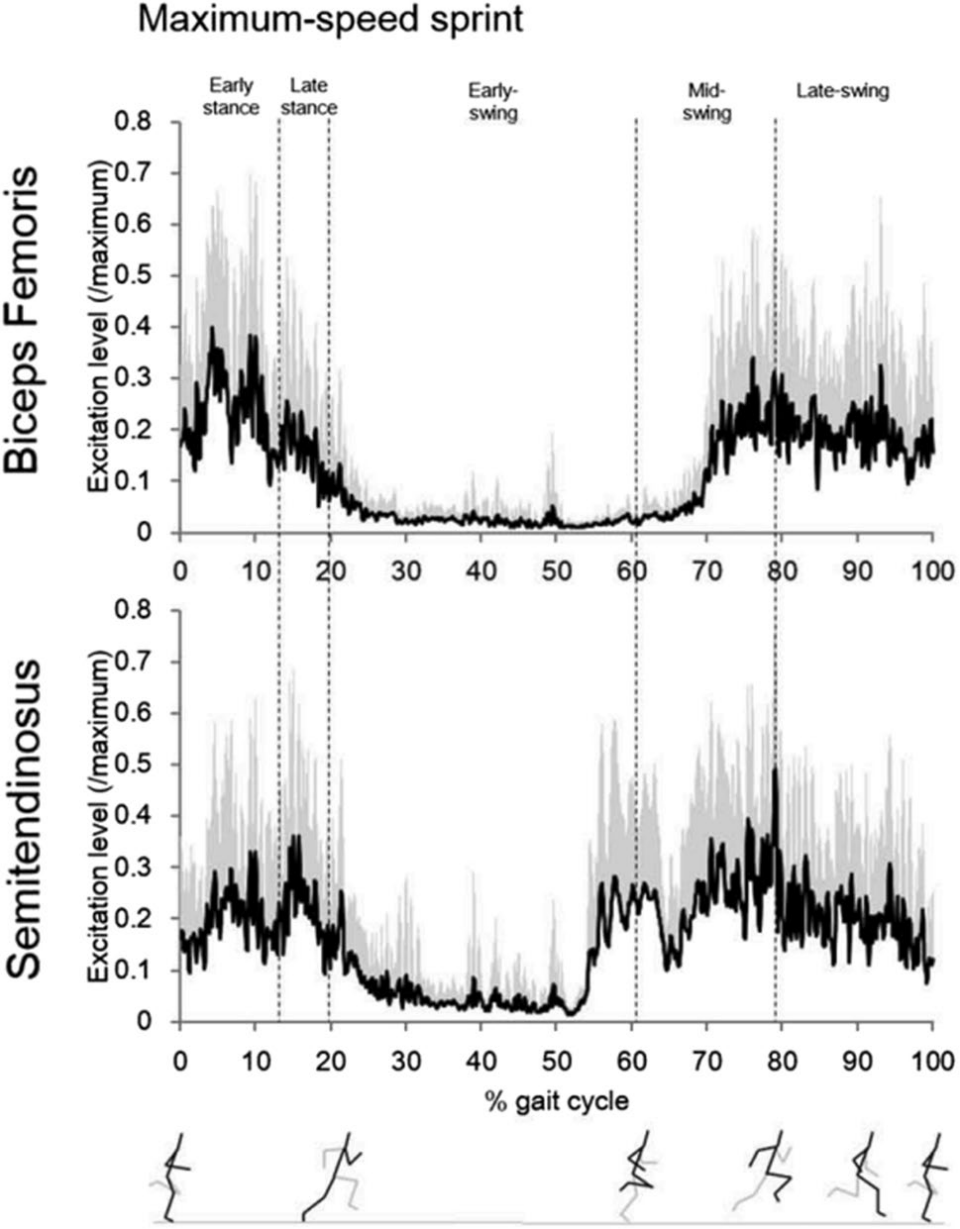
Mean (black) and standard deviation (grey) of the normalised electromyography (EMG) signals of the hamstrings during maximum-speed sprinting. Adapted from Higashihara et al. [177] with permission

In the studies reporting that the hamstrings are not active for the entire sprint cycle, similar findings are presented [6, 15, 177, 178]. Selecting the studies of Higashihara et al. [177] (Fig. 6) and Schache et al. [14] as references, it is observed that electrical signalling to the medial hamstrings begins near the end of the early-swing phase (starting at ~ 50–55% of the gait cycle), while electrical signalling to the lateral hamstrings begins approximately halfway through mid-swing (~ 65–70% of the gait cycle) [177]. When ignoring any potential effects of R-EMD, the medial hamstrings have a relatively lengthy 103.5–113 ms time period for MTU slack to be removed and for tensions to be generated prior to late-swing (a period within which considerable hamstring lengthening occurs) [14, 15, 25]. However, the engagement of the lateral hamstrings during late-swing is more ambiguous. If a relatively short A-EMD of 24 ms is assumed [184, 185], the biceps femoris will be generating active tensions during the later portions of mid-swing and all of late-swing. If we assume a relatively long EMD of 127 ms [180], the lateral hamstrings would not produce tension for the entire late-swing, a seemingly implausible suggestion given the weight of evidence indicating substantial hamstring involvement in this phase [6, 7, 9, 14, 15, 25]. If the effects of R-EMD are considered, tensions may never completely dissipate, which would prevent the reintroduction of slack into the MTU and ensure that all actions are performed under “active” conditions.

Going forward, future research implementing new, more sophisticated technologies, such as high-density EMG, may provide more precise understandings of hamstring muscle activation patterns when sprinting. Regardless, due to the magnitude of uncertainty when attempting to infer the onset and offset of muscle tensions from existing EMG and EMD data, investigation of other available data, such as data pertaining to hamstring kinematics and kinetics, may provide greater clarity regarding potential hamstring functioning and engagement while sprinting.

## Hamstring Kinematics and Kinetics during the Swing Phase of Sprinting

**Fig. 7:**
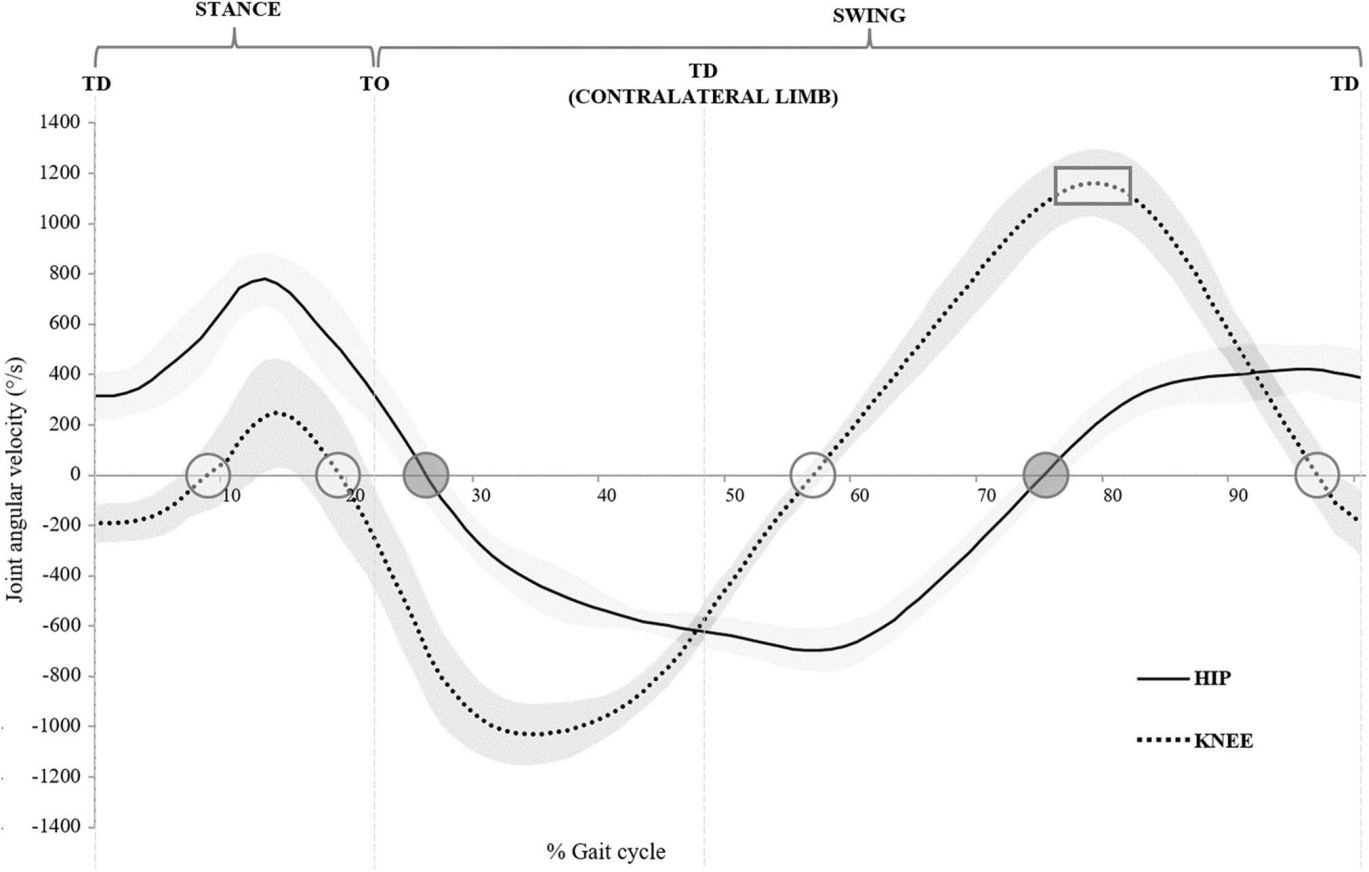
Joint angular velocity profile of a full gait cycle of the knee and hip joints. Standard deviation is represented in grey. Data taken from ten international and national level sprinters with an average peak sprint velocity of 10.23 m/s. TD, touchdown; TO, toe off. Adapted from Sides [12] with permission

Understanding the joint and limb kinematic profiles of the lower limbs during the swing phase of sprinting provides valuable insights into potential hamstring muscle behaviour during this phase. Velocity–time curves of the hip and knee joints while sprinting (Fig. 7) [12] are particularly useful. Initially, if a definition of contraction modality based on the movement of bony attachments is adopted, it can be concluded that an isometric contraction does not exist. Indeed, as depicted in Fig. 7, the hip and knee joint angular velocities do not cross zero on the *x*-axis at the same time, demonstrating that movement is occurring at either the hip or knee joint at any given point in time. Additionally, the transition from joint extension to flexion at both the knee or hip joint is so short in duration that the data frequency used (300 frames per second) by Sides et al. [12] to capture angular velocity did not display a zero velocity point, rendering any potential isometric instant as imperceptible. However, as noted previously, definitions based on bony attachments are misleading in relation to muscle function, and while the bony attachments may be in a constant state of motion, it certainly remains possible that the movement occurring in late-swing is primarily modulated by stretch of the tendons, as has been considered [15, 16]. Accordingly, deeper insights into the kinematic profiles of the lower limbs, in conjunction with hamstring kinetics, are required to provide greater insights into hamstring function.

During the mid-swing phase, the biarticular (with the exception of BFsh) hamstring muscles are stretched due to the hip being in a flexed position while the knee is extending [11, 14, 15]. The hip joint then begins to extend in this phase, with hip extension velocity increasing in mid- and late-swing while the knee joint continues to extend (Fig. 7) [12]. Notably, in the sagittal plane, an exceptionally high peak knee joint extension velocity occurs (> 1000°/s) near the end of mid-swing, just prior to late-swing. A rapid deceleration of the knee joint occurs thereafter. This sudden decrease in knee joint extension velocity (occurring at ~ 78% of the sprint gait cycle [Fig. 7]) complements existing EMG data and is indicative of an active braking role of the hamstrings required to rapidly decelerate the knee joint. Specifically, in this phase the hamstrings are required to withstand forces as high as ten times body weight [9, 26], which is seemingly beyond the maximal isometric force production capabilities of the hamstrings [9]. Indeed, in elite male sprinters, the maximal muscle torque at the knee joint during late-swing is reportedly ~ 1.5 times the maximal voluntary isometric knee flexion torque that these athletes could actively produce [9]. In a separate study by Alt et al. [26], peak knee flexor torques in late-swing also exceeded the torques produced during isokinetic eccentric testing. When the management of these large mechanical loads is considered in the context of the hamstrings being lengthened as the knee is extended [6, 7, 14, 15, 25], stretching to maximum or near maximum lengths during late-swing [8, 11, 15, 25], an eccentric braking role for the hamstrings certainly appears likely. Such a role seems necessary not only to decelerate the knee joint and cope with the large forces (beyond isometric capacity) experienced when sprinting [6, 7, 9, 14, 188, 189], but also to maintain control of the movement and simultaneously meet the lengthening demands placed on the hamstrings during the sprint cycle [6, 7, 14].

## The Importance of Comprehensive Evidence-Based Hamstring Training Frameworks

Theories surrounding functional replication and hamstring contraction modality when sprinting have served as a primary justification for the recommendation of certain hamstring-specific training and injury prevention exercises [16, 17]. For example, proponents of the theory that the hamstrings contract isometrically while sprinting propose that the hamstrings should be “trained in a way whereby the CE (interpreted as the muscle fascicles) remains isometric, while the SEE (interpreted as the tendon and other connective tissue) stretches and recoils rather than using eccentric exercises.” [16]. This suggestion is flawed for a number of reasons. Firstly, as thoroughly outlined in Sect. 2, this is a simplified interpretation of muscle function that neglects the viscoelastic properties of muscle fibres. Secondly, although specificity is an important training principle, the evidence supporting that any of the proposed “isometric” hamstring exercises [17], and many other hamstring-specific exercises, replicate hamstring MTU functioning while sprinting is minimal. Most importantly, however, is that it is unclear as to why replication forms the exclusive qualifying characteristic for hamstring exercise selection. Adaptive benefits may potentially be derived from a variety of exercises that provide different training stimuli, and that do not necessarily replicate hamstring MTU functioning when sprinting. Rather, a more rigorous approach to hamstring training is to select hamstring-specific exercises on the basis of rigorously developed evidence-based frameworks surrounding the specific stimulus provided by the exercise, the accompanying adaptations elicited by the exercise, and the causal effects of these adaptations on hamstring functioning and injury risk mitigation while sprinting. This is important as muscle adaptation can be counterintuitive. For example, if the aim is to reduce active fascicle and fibre strains to restrict muscle damage when sprinting, and therefore encourage the hamstring muscles to function more closely to “isometric” (particularly at long muscle lengths), eccentric exercises may provide the best means to achieve this. Indeed, some of the training adaptations induced by eccentric exercises include a shift in the force–length profile of a muscle towards longer lengths [162, 190], and a reduction in fascicular strains [151]. Along with increases in muscle strength, one proposed mechanism through which eccentric training achieves this includes an increase in fascicle lengths [191]. Increases in fascicle length in response to eccentric training are commonly purported to occur as a result of an increase in the number of sarcomeres in-series [190, 192], which is also understood to explain the repeated bout effect [190, 192]. By increasing the number of sarcomeres in-series, each individual sarcomere may experience reduced strains due to more optimal sarcomere lengths of functioning at longer fibre lengths, and the distribution of strain across a greater number of sarcomeres [190, 192]. This preserves the muscle during sustained activity by lessening the eccentrically induced muscle damage experienced [190, 192], which is an important consideration for athletes involved in repeated sprint activities or when relatively short recovery times are imposed on an athlete. However, it is important to note that increases in fascicle length may present for reasons other than the addition of sarcomeres in-series [193], with some other potential explanations including changes in resting sarcomere length, decreases in resting muscle tension, increases in end-point tension (e.g., through increased stiffness of tendon/aponeurosis) or the longitudinal translocation of myofibrils or whole fibres within the muscle [193–195]. Regardless, the adaptations derived from eccentric hamstring interventions may provide many benefits for athletes required to sprint, from potentially reducing the risk of hamstring injury [196–198] to improving both lower-limb mechanics and overall sprint performance [199].

Various biomechanical parameters observed when sprinting such as contraction mode, knee and hip joint configurations, range of motion and joint moments can, to some extent, be reproduced during targeted hamstring training [200]. However, muscle function is intricate and dependent upon complex interactions between musculoskeletal kinematics and kinetics [201], muscle activation patterns and the neuromechanical regulation of tensions and stiffness [59, 201–204], and loads applied by the environment [201, 205, 206], among other important variables. Accordingly, the extent to which isolated hamstring exercises, whether concentric, isometric or eccentric, replicate hamstring MTU function when sprinting is highly questionable. To emphasise, it has recently been demonstrated that across a large range of exercises utilising the hamstrings, none of the exercises activated the hamstring muscles more than an average of 60% of the maximal activation exhibited during top-speed sprinting [207]. In addition, the exceptionally high angular velocities experienced at the hip (> 700°/s) and knee (> 1000°/s) joints (Fig. 7) are also far beyond those experienced during traditional resistance training exercises [26, 142]. This is not simply a question of electrical signalling magnitude and joint angular velocities, however. The specific mechanical demands influencing hamstring MTU function while sprinting (e.g. specific timings and combinations of hip and knee joint angles, velocities, accelerations and moments, magnitude and rates of muscle loading, required combinations of muscle and tendon lengths and length changes, muscle and tendon stretch and shortening velocities and accelerations, etc.) are unique and rapidly fluctuating throughout the sprint cycle in a finely regulated manner that is highly specific to this activity. Additionally, the functioning of each individual hamstring MTU (e.g. muscle signalling and activation pattern, neuromechanical regulation of tension and stiffness, muscle–tendon dynamics including magnitude and rates of length changes and contraction velocities, etc.) to cope with these rapidly fluctuating demands is similarly anticipated to be unique to this particular locomotive task. If the aim is to replicate hamstring muscle–tendon function when sprinting, this is likely an unattainable goal for any isolated hamstring exercise. Reasonably, the best way to achieve such a goal is to sprint, with hamstring loading perhaps best being regulated by varying the sprint conditions (e.g., sprint intensity, additional weight, sleds, etc.). However, it is important to note that mechanical loads and hamstring functioning may still vary substantially when there are seemingly small alterations in sprint execution, as has been observed between maximal-effort sprinting and high-speed running (e.g., 80% of maximum) [6, 189]. This warrants deliberation. In some sporting contexts such as football (soccer), most hamstring injuries occur when players are sprinting at above 80% of maximum [208]. Regardless, replication of hamstring functioning when sprinting should not be the exclusive qualifying characteristic for targeted hamstring exercise selection. A variety of hamstring exercises that provide different training stimuli can potentially offer adaptive benefits that are valuable to hamstring functioning, athletic performance, rehabilitation and injury risk mitigation in athletes required to sprint. This is an important consideration, especially when sprinting is not an option due to injury or other reasons.

Ideally, the adoption of specific hamstring-focused exercises for either athletic performance or injury prevention should not be grounded in unvalidated claims of replication of muscle–tendon function. Rather, the selection of hamstring-specific exercises should be guided by comprehensively constructed evidence-based frameworks surrounding the specific stimulus provided by the selected exercise, the resulting adaptations elicited by the exercise, and the causal effects of these exercises and their accompanying adaptations on hamstring functioning, sprinting performance and injury risk mitigation when sprinting. The development of frameworks and models constitutes an important part of the research process [209, 210]. These tools map out complex systems (in this case hamstring function, training and injury) by aligning relevant variables within a coherent structure of justification [209, 210], highlighting the specific contributions to and causal roles of selected variables in a particular complex system and outcome of interest. Notably, two evidence-based frameworks for strengthening exercises to prevent hamstring injury have already been published in the literature [200, 211]. Within the most recent framework presented by Bourne et al. [211], differences in the stimulus provided, acute responses and chronic adaptations elicited by different training exercises are explored, which is most appropriate. Additionally, evidence surrounding the relationships between hamstring strength and muscle architecture, morphology and function, and the primary response of interest (injury) are also presented. However, some notable limitations of this framework include that a small number of potential causal variables were explored (primarily strength and architecture), and an explicit visual framework mapping out the positioning of these variables within the complex system of hamstring function and injury is not provided. Consequently, the examined variables were primarily explored in isolation.

Future frameworks surrounding hamstring training and injury should integrate the effects of training on a broader range of potential causal variables contributing to hamstring function and injury. Some examples include the effects of training on the neuromechanical regulation of hamstring muscle stiffness and tensions, the mechanical characteristics of surrounding connective tissues such as the hamstring tendons and aponeuroses, as well as variables influencing hamstring loading, such as sprint mechanics. Further, these frameworks should position the examined variables within a clear causal structure so that the interrelationships between variables can be observed, and the contributions of these variables to the overall system can be investigated and understood. This will facilitate the amalgamation of lab-based and mechanistic research with injury outcome-based research to provide a more comprehensive understanding of hamstring injury prevention and training. This will also assist in making explicit current gaps in the scientific literature, providing research direction to the sports science and medicine community by clarifying which aspects of hamstring training, adaptation and injury exhibit a strong evidence base, and which lack evidence and require further research.

## Future Research Directions

Currently, there remains a strong dependency on theory to understand hamstring function when sprinting. While the currently available data are useful for guiding theory development relating to hamstring function when sprinting, more precise and detailed investigations are still required to uncover the specific behaviour of the hamstring muscle fibres and tendons during this particular task. A more thorough understanding of the hamstrings may have important implications for understanding athletic injury mechanisms, as well as for informing the development and implementation of athletic training, rehabilitation and injury risk mitigation protocols. To advance research understandings in the area of muscle–tendon mechanics and hamstring functioning during sprinting, exploration of some potential avenues for more precise investigations of hamstring function when sprinting may be beneficial. A brief synopsis of three potential methods is provided below.

### Sonomicrometry

One such method for assessing MTU behaviour used within the literature is sonomicrometry [212–214]. Sonomicrometry permits the assessment of muscle and fascicle length changes by embedding miniature ultrasonic crystals into muscles and measuring the distance between these crystals on the basis of the speed of acoustic signals. Such an approach has been used in a variety of animals to assess muscle–tendon behaviour during various movement tasks [212, 214]. However, the invasive nature of embedding ultrasonic crystals into human tissue raises ethical concerns that restrict its application. Accordingly, other techniques such as computational musculoskeletal modelling provide a more ethical alternative for humans.

### Computer Modelling and the Hill Model

Modelling of human muscle provides a potential non-invasive avenue for assessing muscle–tendon behaviour. The most commonly utilised model in biomechanics and human movement science to actuate musculoskeletal models in simulations of human movement is the Hill muscle model (Table 1) [67]. Indeed, Hill-type models have typically been used to explore hamstring function when sprinting [6, 7, 14]. While such models hold value in a research context, it is important to note that these models function on a number of assumptions and there remains a series of important limitations. For example, Hill-type models typically assume uniform mechanical strain distributions along muscle fibres and tendons [15, 19], which is inaccurate [43, 215], and they also do not appropriately account for velocity transients in strain trajectories [201]. Perhaps most importantly, muscle function is complicated, and Hill-type models still do not accurately predict muscle forces under dynamic conditions [20–22]. To improve upon current musculoskeletal models, and to better understand in vivo hamstring functioning during locomotion, future computational models need to appropriately account for the viscoelastic properties of muscle, e.g. stiffness and damping, which are tuned with activation [202, 203]. In addition, these models also need to account for the decoupling between electrical signalling and force production, and the interaction between strain trajectories and muscle activation patterns [201]. Indeed, this particular interaction has important implications for muscle forces under dynamic conditions [201]. While computational models hold promise as a valuable approach to understanding hamstring muscle–tendon behaviour when sprinting in the long term, current musculoskeletal models still have many limitations.

### Ultrasound

Considering that computational models attempting to model muscle–tendon function and energetics during dynamic activities still have many limitations, ultrasound may provide the most viable avenue for accurately assessing and understanding hamstring muscle–tendon behaviour while sprinting. Ultrasound has commonly been used in humans to estimate muscle and fascicle strains during various dynamic locomotive activities [90]. However, ultrasound techniques for assessing muscle–tendon function often adopt highly extrapolative methods [41], while the movement intensities of sprinting coupled with the location of the hamstrings make assessing this muscle group during this particular task especially challenging. Accordingly, innovative approaches adopting ultrasound technology, such as those that reduce device bulkiness [216, 217] and provide extended fields of view [41], or perhaps alternative approaches utilising different technologies, are still required to permit the accurate assessment of hamstring muscle–tendon function when sprinting.

## Conclusion

Muscles perform a variety of functions during locomotion, acting as motors, brakes, springs and struts. During locomotion, two primary models of MTU functioning have been theorised: an efficiency model and a power model. The theory that the hamstrings function isometrically while sprinting is primarily grounded in an efficiency-based spring-driven model of MTU functioning. In this mode of functioning, propositions supporting the prioritisation of isometric MTU work loops are often centred around the unsupported assumption that isometric work loops offer metabolic savings during locomotion compared with muscular stretch–shorten cycles. It follows that, during spring-driven MTU functioning, muscular stretch–shorten cycles are common and some active lengthening is typically experienced. This provides a series of functional benefits through force enhancement-related mechanisms. Regardless, sprinting is a maximal-effort, sustained high-velocity activity that exposes the hamstrings to large mechanical loads that are seemingly beyond isometric capacity. It is therefore anticipated that, when sprinting, the hamstring muscles adopt a model of functioning that has some reliance upon active muscle lengthening and muscle actuators. Despite this, considering the prominent connective tissue structures, e.g. tendons and aponeuroses, that can be observed in the hamstrings, there is anticipated to be considerable tendon contributions to hamstring function when sprinting, with the hamstring tendons serving as a mechanical buffer that reduces fascicular strains, and potentially as a spring that stores and releases elastic energy.

Currently, the majority of existing evidence alludes to the hamstring muscles functioning as an eccentric energy-absorbing brake that decelerates knee extension and tolerates high mechanical loads in the late-swing phase of sprinting. However, the architecture of the hamstrings varies between individual muscles. Relative to one another, the longer, thinner muscles of semitendinosus and BFsh appear better suited for large fascicular excursions, while BFlh and semimembranosus display relatively short fascicles and long tendons, and therefore appear better suited for spring-driven locomotion. It is therefore anticipated that various combinations of spring-, brake- and motor-driven functioning exist across the hamstring muscles during the sprint cycle, with muscle–tendon behaviour and work distributions differing between each individual hamstring muscle in a manner that is reflective of their architectural arrangement and activation patterns. Currently, a method to accurately assess muscle behaviour and the distribution of work between muscle and tendon within the hamstrings when sprinting does not exist, and accordingly, the precise functioning of each individual hamstring MTU during this particular task remains uncertain.

Finally, muscle function is intricate and dependent upon complex interactions between a number of variables including musculoskeletal kinematics and kinetics, muscle activation patterns and the neuromechanical regulation of tensions and stiffness, and loads applied by the environment. Accordingly, hamstring MTU functioning during sprinting is anticipated to be unique to this specific activity. The extent to which isolated hamstring exercises replicate hamstring functioning when sprinting is questionable, irrespective of contraction modality. However, adaptive benefits that are useful for athletes required to sprint can likely be derived from a variety of hamstring-specific exercises. It is therefore proposed that the adoption of hamstring-specific exercises should not be founded on unvalidated claims of replicating hamstring function when sprinting. Rather, a more rigorous approach is to select hamstring-specific exercises on the basis of thoroughly constructed evidence-based frameworks surrounding the specific stimulus provided by the exercise, the accompanying adaptations elicited by the exercise, and the effects of these adaptations on hamstring functioning and injury risk mitigation while sprinting.

## Data Availability

Not applicable.
